# Number of Tumor Foci as a Risk Factor for Recurrence in Papillary Thyroid Carcinoma: Does It Improve Predictability?

**DOI:** 10.3390/cancers14174141

**Published:** 2022-08-26

**Authors:** Hyungju Kwon, Woosung Lim, Byung-In Moon

**Affiliations:** Department of Surgery, Ewha Womans University Medical Center, 1071 Anyangcheon-ro, Yangcheon-Gu, Seoul 07985, Korea

**Keywords:** papillary thyroid carcinoma, multifocality, number of tumor foci, recurrence

## Abstract

**Simple Summary:**

Thyroid cancer is one of the most common cancers worldwide, and papillary thyroid carcinoma (PTC) comprises over 80% of all thyroid cancers. About 30% of patients with PTC have multifocality, which is useful for recurrence prediction. However, recent studies have suggested that the number of tumors, total tumor diameter, and bilaterality are more powerful predictors of recurrence than multifocality. Herein, we evaluated the effect of these factors on the predictability of recurrence in patients with PTC. Our study of 1288 patients confirmed that the number of tumors, total tumor diameter, and bilaterality could be independent predictors of recurrence even though they did not offer better predictability of recurrence than the prediction model using multifocality. Therefore, a simpler, multifocality-based prediction model would be sufficient for predicting recurrence in patients with PTC.

**Abstract:**

Multifocality in papillary thyroid carcinoma (PTC) increases the risk of recurrence. Some recent studies have suggested that multifocality-related parameters, such as the number of tumor foci, total tumor diameter (TTD), and bilaterality, are more useful for predicting recurrence than multifocality. However, it is still unclear if these factors can improve the accuracy of the recurrence prediction model. Between 2012 and 2019, 1288 patients with PTC underwent total thyroidectomy at Ewha Womans University Medical Center. The 5-year disease-free survival rate was 91.2% in patients with >3 tumor foci, 95.1% with 3 foci, and 97.6% with 2 foci; conversely, those with a unifocal tumor showed a 5-year recurrence-free survival rate of 98.0%. Cox proportional hazards analysis indicated that the number of tumor foci (HR for >3 foci, 3.214; HR for 3 foci, 2.473), bilaterality (HR, 2.530), or TTD (HR for >3 cm, 5.359; HR for 2–3 cm, 3.584) could be an independent predictor of recurrence. However, models using the number of tumor foci, bilaterality, and TTD did not show better overall predictability of recurrence than models based on multifocality. In conclusion, a simpler prediction model based on multifocality may be sufficient.

## 1. Introduction

Thyroid cancer is one of the most common malignancies worldwide, and its incidence has steeply risen over the last three decades [1,2]. There were 586,202 newly diagnosed thyroid cancer cases in 2020, representing 3.0% of all cancer patients [1]. Papillary thyroid carcinoma (PTC) constitutes over 90% of all thyroid cancers in Korea and usually has a favorable prognosis. Nonetheless, up to 35% of patients experience considerable disease progression of thyroid cancer, including regional recurrences or distant metastases [3,4]. Many investigators have tried to identify these high-risk populations from the patients with excellent outcomes [5,6]. Clinicopathological features, such as multifocality, tumor size, extrathyroidal extension (ETE), and lymph node (LN) metastases, have been meticulously investigated to predict recurrence or disease-specific mortality.

Multifocality in thyroid cancer is defined as tumors with two or more distinct locations within the thyroid gland. Multifocal tumors are commonly found in PTC and have a prevalence rate of 18–87% among the cases reported in the literature [7]. Multifocality has been considered a predictive factor for PTC progression [7]. Many studies indicate that multifocality is associated with high-risk features of PTC, such as aggressive histologic subtype, ETE, regional LN involvement, and distant metastases [8,9]. A meta-analysis in 2021 also concluded that multifocality could be an independent predictor of recurrence [5]. Several researchers further demonstrated that multifocal PTCs would decrease overall and cancer-specific survival [10].

Recent studies have investigated various multifocality-related morphological parameters, such as number of tumor foci, total tumor diameter (TTD), and bilaterality, to determine tumor behavior in multifocal PTC [11,12,13,14]. Several investigators demonstrated that the recurrence rate could proportionately increase with an increasing number of tumor foci [12]. Other researchers suggested that TTD better assesses the aggressiveness of the tumor [13]. Some reports suggested that bilaterality was more strongly predictive of a poor prognosis of PTC than unilateral multifocality [14]. However, it is still unclear whether number of tumors, TTD, and bilaterality improve the predictability of recurrence in patients with multifocal PTC. Therefore, in the present study, we investigated the impact of these factors to improve the overall predictability of recurrence in patients with PTC.

## 2. Materials and Methods

The institutional review board of the Ewha Womans University Medical Center approved this retrospective cohort study (Approval No. 2022-07-032), and waived the documentation of informed consent. We included 1288 consecutive patients with PTC, who underwent total thyroidectomy between January 2012 and December 2019. All surgical procedures were performed by three experienced surgeons (H.K., W.L., and B.-I.M.) who had annually performed >100 thyroidectomies, respectively. Medical records of all patients were reviewed for clinicopathological data. The American Joint Committee on Cancer 8th edition was applied for pathologic tumor-node-metastasis (TNM) staging. Patient demographics and tumor characteristics data pertaining to tumor size, ETE, total number of tumors, LN metastasis, resection margin statue, and coexisting Hashimoto thyroiditis were collected. Patients with incomplete data were excluded. Patients were also excluded if they had distant metastasis before operation. The primary end point of this study was 5-year disease-free survival (DFS).

R 4.1.2 (R Development Core Team, Vienna, Austria) and SPSS Statistics 23.0 (IBM Corp., Armonk, NY, USA) were used for data analyses. Student’s *t*-tests were used to compare continuous variables. Dichotomous variables were compared using chi-squared tests. We performed 1:1 propensity score matching to minimize biases from possible confounders, such as age, tumor size, ETE, LN metastasis, and margin involvement. DFS was assessed using the log-rank test and Kaplan–Meier plots. Cox proportional hazards model was used to formulate prediction scores based on the regression coefficients. The area under the receiver operating curve (AUROC) was used to evaluate the relative predictability of prediction scores. Time-dependent AUROC analysis was used to compare AUROCs from each prediction model. A p-value less than 0.05 was considered statistically significant. A *p*-value less than 0.05 was considered statistically significant.

## 3. Results

### 3.1. Clinicopathological Characteristics of 1288 Patients with PTC

Table 1 shows the baseline characteristics of the included patients. The mean age at the time of initial thyroidectomy was 47.4 ± 11.7 years (interquartile range [IQR] 39–55 years), and 1126 of the patients (87.4%) were women. The follow-up period was 6.4 ± 3.3 years (IQR 4.4–8.7 years). A total of 787 (61.1%) patients had a unifocal tumor, whereas 322 (25.0%) patients had 2 tumor foci, 114 (8.9%) had 3 tumor foci, and 65 (5.0%) had ≥4 tumor foci. Primary tumor size was 1.0 ± 0.7 cm, and TTD was 1.3 ± 0.9 cm with a median diameter of 1.1 cm (IQR 0.7–1.6). Bilaterality was observed in 338 (26.2%) patients. Recurrence occurred in 37 patients (2.9%).

### 3.2. Comparison of Recurrence Rates According to the Number of Tumor Foci

Table 2 shows a comparison of patient characteristics according to the number of tumor foci. Patients with ≥4 tumor foci (*n* = 65) were classified as belonging to the TF4 group, whereas those with 3 foci (*n* = 114), 2 foci (*n* = 322), and 1 focus (*n* = 787) were classified as belonging to TF3, TF2, and TF1 groups, respectively. A larger number of tumor foci was associated with a larger TTD (*p* < 0.001), microscopic ETE (*p* = 0.002), and LN metastasis (*p* = 0.002). Recurrences were found in 5 (7.7%) patients in the TF4 group, 6 patients (5.3%) in the TF3 group, 10 (3.1%) in the TF2 group, and 16 (2.0%) in the TF1 group (*p* = 0.020). The 5-year DFS was 91.2% in TF4, 95.1% in TF3, and 97.6% in TF2 groups whereas the TF1 group showed a 5-year DFS of 98.0% (*p* = 0.013).

### 3.3. Comparison of Recurrence Rates after Propensity Score Matching

**Figure 1:**
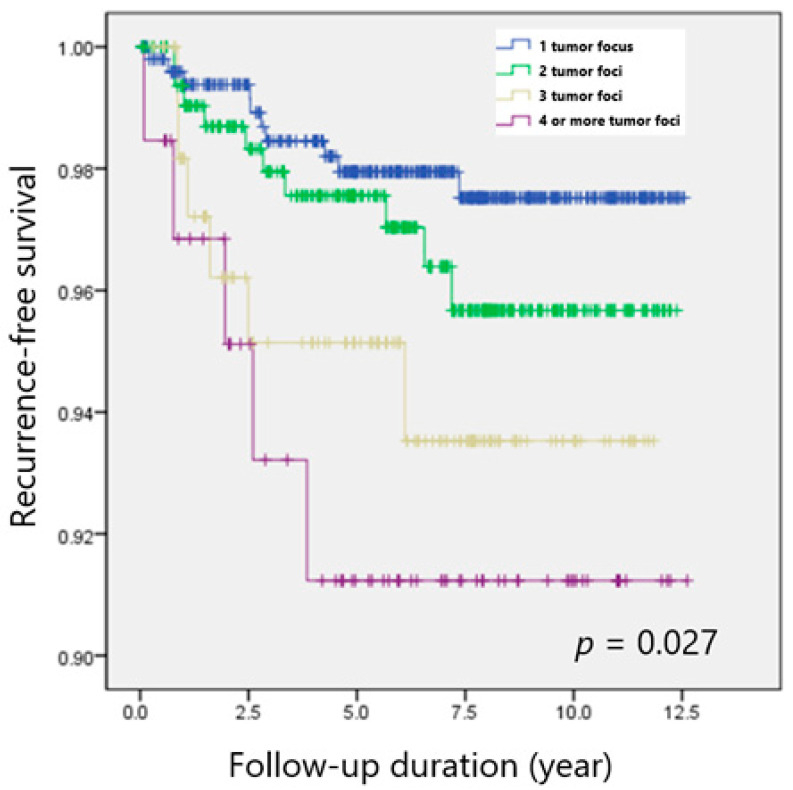
Recurrence-free survival according to the number of tumor foci in patients with PTC.

Table 3 shows a clinicopathological comparison among groups after 1:1 propensity-score matching. Clinicopathological features, including microscopic ETE and LN metastasis were comparable in the matched cohorts, whereas TTD significantly differed among the groups. Both the overall recurrence rate (*p* = 0.039) and 5-year DFS (*p* = 0.027) still showed significant difference among groups after propensity score matching (Figure 1). 

### 3.4. Comparison of Predictability of Recurrence of Various Prediction Models

Cox proportional hazards regression analyses were performed to determine significant predictors of recurrence. Multifocality (HR, 2.404; 95% CI, 1.125–5.135), bilaterality (HR, 2.530; 95% CI, 1.226–5.219), the number of tumor foci (HR, 3.214 for ≥4 tumor foci; 95% CI, 1.115–9.265), and TTD (HR, 3.584 for 2–3 cm; 95% CI, 1.345–9.548; HR, 5.359 for >3 cm; 95% CI, 1.322–21.718) were found to be independent predictors of recurrence.

Using these factors, we formulated prediction scores using Cox hazards regression models (Table 4). Predictability as measured by the AUROC of each prediction model indicated that models based on the number of tumors (AUROC of 0.718) and bilaterality (AUROC of 0.719) were more powerful than that based on multifocality (AUROC of 0.716). However, these models based on the number of tumors (*p* = 0.946) and bilaterality (*p* = 0.887) did not substantially improve the overall predictability of recurrence. The model using TTD (AUROC of 0.631) also showed no significant improvement in predictability of recurrence (*p* = 0.217).

## 4. Discussion

The present study revealed that although an increasing number of tumor foci was associated with a higher risk of recurrence in PTC, it did not improve the accuracy of the recurrence prediction model. Multifocal PTCs may have distinctive characteristics according to the number of tumor foci, thus resulting in different recurrence rates [15]. Previous studies suggested that ≥3 tumor foci was associated with an increasing risk of recurrence compared to 2 foci [16]. Lin et al. further demonstrated that recurrence rates would increase up to 45.8% in proportion to the number of tumor foci [12]. This is partly because multifocal tumors can be the result of intrathyroidal spread from one tumor focus, which may represent the aggressiveness of the tumor [15,17].

The number of tumor foci is associated with several high-risk characteristics of PTC [18,19]. Some researchers indicated that the total number of tumors was associated with an aggressive histologic subtype of PTC or a higher American Thyroid Association risk of recurrence [8,9]. Feng et al. suggested that patients with a higher number of tumor foci had a greater risk of ETE, vascular invasion, and LN metastasis [16]. Furthermore, a recent meta-analysis showed that multifocal PTCs were associated with an increased risk of tumors larger than 1 cm, ETE, or LN metastases [7]. We also demonstrated that the number of tumor foci was associated with microscopic ETE and LN metastasis. As LN metastasis and ETE could increase the risk of recurrence, we conducted 1:1 propensity score matching to minimize biases and compared DFS according to the number of tumor foci.

In the present study, we identified the number of tumors, bilaterality, and TTD as potential independent predictors of recurrence in patients with PTC. DFS decreased proportionally with an increasing number of tumor foci; however, the risk of recurrence of tumors with two foci and unifocal tumors was comparable. A recent meta-analysis also concluded that patients with ≥3 tumor foci had a higher risk of recurrence than those with 2 tumor foci, and 2 tumor foci showed recurrence rates similar to unifocal tumor [5]. When we built a prediction model using the number of tumors, the predictability of recurrence (AUROC, 0.718) was comparable to that using multifocality (AUROC 0.716; *p* = 0.946). This result suggests that a model using multifocality is sufficient for prediction of recurrence.

There are conflicting views on whether bilaterality increases the risk of recurrence. Several researchers reported bilaterality as not a prognostic factor for the recurrence of PTC [20,21,22,23]. In contrast, Qu et al. reported that bilateral multifocality was associated with the recurrence of PTC [14]. Wang et al. also reported that patients with bilateral PTC had shorter DFS than those with unilateral multifocal tumors [24]. Ding et al. found that bilaterality increased the risk of recurrence by 1.6 times [25], and, consistent with their findings, bilaterality raised the risk of recurrence in our study (HR, 2.503; 95% CI, 1.226–5.219). Prediction model using bilaterality had the highest predictability with an AUROC value of 0.719; however, it showed no significant improvement in predictability over the model using multifocality (*p* = 0.887).

Notably, TTD has been considered as a marker of tumor aggressiveness. Manso et al. suggested that TTD better represents the tumor burden in multifocal PTC and that DFS is significantly shorter in patients with a large TTD [26]. TTD can also be considered a risk factor even in patients with small PTCs, including papillary thyroid microcarcinoma (PTMC). Zhao et al. reported that TTD predicted LN metastasis more accurately than primary tumor size in patients with PTMC [27]. Hitu et al. further suggested that TTD was an independent predictor of metastatic PTMC [11]. We also demonstrated that TTD was associated with an increased risk of recurrence; however, our prediction model using TTD did not show improved predictability over the model using multifocality.

This study has some limitations. First, this was a retrospective cohort study, making it prone to selection bias. Second, most patients enrolled in the present study had the classical subtype of PTC. Some variants like the tall cell variant carry the BRAF mutation, which may increase the risk of recurrence. Therefore, validation for the impact of number of tumor foci is required in other subtypes of PTC along with BRAF mutation analysis. Finally, long-term prognoses, including mortality, was not evaluated in the present study. A follow-up period of 6.4 years was not enough for investigating cancer-specific survival. Further studies are warranted to address these issues.

## 5. Conclusions

Although the number of tumor foci, bilaterality, and TTD were independent predictors of recurrence, they did not improve the predictability of recurrence. A simpler prediction model based on multifocality may be sufficient.

## Figures and Tables

**Table 1:** Comparison of clinicopathological characteristics between patients with multifocal PTCs and those with unifocal tumor.

Characteristics	
Age (years)	47.4 ± 11.7 (IQR 39–55)
Female sex	1126 (87.4%)
Pathologic features	
Number of tumor foci	
1 (unifocal)	787 (61.1%)
2	322 (25.0%)
3	114 (8.9%)
≥4	65 (5.0%)
Primary tumor size (cm)	1.0 ± 0.7 (IQR 0.6–1.2)
Total tumor diameter (cm)	1.3 ± 0.9 (IQR 0.7–1.6)
Microscopic ETE	787 (61.1%)
Bilaterality	338 (26.2%)
LN metastasis	
N0	743 (57.7%)
N1a	545 (42.3%)
Margin involvement	43 (3.3%)
Coexisting HT	363 (28.2%)
Postoperative management	
^131^I remnant ablation	568 (44.1%)
^131^I dose (mCi)	134.1 ± 35.0 (IQR 100.0–150.0)
Follow-up period (years)	6.4 ± 3.3 (IQR 4.4–8.7)
Recurrence	37 (2.9%)

PTC, papillary thyroid carcinoma; ETE, extrathyroidal extension; LN, lymph node; HT, Hashimoto thyroiditis.

**Table 2:** Comparison of clinicopathological characteristics among patients with PTC according to the number of tumor foci.

Characteristics	TF4 (*n* = 65)	TF3 (*n* = 114)	TF2 (*n* = 322)	TF1 (*n* = 787)	*p*-Value
Age (years)	49.1 ± 10.7	48.1 ± 11.9	47.7 ± 11.2	47.0 ± 11.7	0.383
Female sex	54 (83.1%)	102 (89.5%)	278 (86.3%)	692 (87.9%)	0.556
Pathologic feature					
Tumor size (cm)	1.0 ± 0.6	1.1 ± 0.7	1.0 ± 0.7	1.0 ± 0.7	0.419
Total tumor diameter					<0.001
0.0–1.0 cm	2 (3.1%)	13 (11.4%)	111 (34.5%)	514 (65.3%)	
1.1–2.0 cm	19 (29.2%)	58 (50.9%)	161 (50.0%)	212 (26.9%)	
2.1–3.0 cm	23 (35.4%)	30 (26.3%)	34 (10.6%)	43 (5.5%)	
>3.0 cm	21 (32.3%)	13 (11.4%)	16 (5.0%)	18 (2.3%)	
Microscopic ETE	47 (72.3%)	79 (69.3%)	212 (65.8%)	449 (57.1%)	0.002
LN metastasis					0.002
N0	25 (38.5%)	59 (51.8%)	181 (56.2%)	478 (60.7%)	
N1	40 (61.5%)	55 (48.2%)	141 (43.8%)	309 (39.3%)	
Margin involvement	3 (4.6%)	3 (2.6%)	18 (5.6%)	19 (2.4%)	0.054
Coexisting HT	18 (27.7%)	31 (27.2%)	85 (26.4%)	229 (29.1%)	0.827
Postoperative course					
^131^I remnant ablation	36 (55.4%)	58 (50.9%)	145 (45.0%)	329 (41.8%)	0.064
^131^I dose (mCi)	154.2 ± 30.2	131.8 ± 47.4	130.3 ± 35.3	133.9 ± 32.2	0.003
Follow-up period (years)	6.3 ± 3.6	6.0 ± 3.2	6.2 ± 3.2	6.6 ± 3.2	0.077
Recurrence	5 (7.7%)	6 (5.3%)	10 (3.1%)	16 (2.0%)	0.020

PTC, papillary thyroid carcinoma; ETE, extrathyroidal extension; LN, lymph node; HT, Hashimoto thyroiditis.

**Table 3:** Comparison of clinicopathological characteristics among patients with PTC according to the number of tumor foci after propensity score matching.

Characteristics	TF4 (*n* = 65)	TF3 (*n* = 114)	TF2 (*n* = 322)	TF1 (*n* = 501)	*p*-Value
Age (years)	49.1 ± 10.7	48.1 ± 11.9	47.7 ± 11.2	46.8 ± 12.0	0.333
Female sex	54 (83.1%)	102 (89.5%)	278 (86.3%)	439 (87.6%)	0.615
Pathologic feature					
Tumor size (cm)	1.0 ± 0.6	1.1 ± 0.7	1.0 ± 0.7	1.0 ± 0.7	0.557
Total tumor diameter					<0.001
0.0–1.0 cm	2 (3.1%)	13 (11.4%)	111 (34.5%)	317 (63.3%)	
1.1–2.0 cm	19 (29.2%)	58 (50.9%)	161 (50.0%)	148 (29.5%)	
2.1–3.0 cm	23 (35.4%)	30 (26.3%)	34 (10.6%)	24 (4.8%)	
>3.0 cm	21 (32.3%)	13 (11.4%)	16 (5.0%)	12 (2.4%)	
Microscopic ETE	47 (72.3%)	79 (69.3%)	212 (65.8%)	332 (66.3%)	0.704
LN metastasis					0.063
N0	25 (38.5%)	59 (51.8%)	181 (56.2%)	255 (50.9%)	
N1	40 (61.5%)	55 (48.2%)	141 (43.8%)	246 (49.1%)	
Margin involvement	3 (4.6%)	3 (2.6%)	18 (5.6%)	19 (3.8%)	0.492
Coexisting HT	18 (27.7%)	31 (27.2%)	85 (26.4%)	143 (28.5%)	0.927
Postoperative course					
^131^I remnant ablation	36 (55.4%)	58 (50.9%)	145 (45.0%)	241 (48.1%)	0.402
^131^I dose (mCi)	154.2 ± 30.2	131.8 ± 47.4	130.3 ± 35.3	135.6 ± 32.7	0.004
Follow-up period (years)	6.3 ± 3.6	6.0 ± 3.2	6.2 ± 3.2	6.7 ± 3.3	0.042
Recurrence	5 (7.7%)	6 (5.3%)	10 (3.1%)	10 (2.0%)	0.039

PTC, papillary thyroid carcinoma; ETE, extrathyroidal extension; LN, lymph node; HT, Hashimoto thyroiditis.

**Table 4:** Predictive models for recurrence in patients with papillary thyroid carcinoma.

Characteristics	Multivariate Analysis		Prediction Model	
	HR (95% CI)	*p*-Value	Area under ROC (95% CI)	*p*-Value
Multifocality	2.404 (1.125–5.135)	0.024	0.716 (0.625–0.807)	Ref.
Bilaterality	2.530 (1.226–5.219)	0.012	0.719 (0.624–0.814)	0.887
Number of tumor foci			0.718 (0.623–0.814)	0.946
2 foci	1.507 (0.679–3.349)	0.314		
3 foci	2.473 (0.953–6.417)	0.063		
≥4 foci	3.214 (1.115–9.265)	0.031		
Total tumor diameter			0.631 (0.525–0.736)	0.217
1.1–2.0 cm	0.890 (0.322–2.461)	0.822		
2.1–3.0 cm	3.584 (1.345–9.548)	0.011		
>3.0 cm	5.359 (1.322–21.72)	0.019		

HR, hazard ratio; CI, confidence interval; ROC, receiver operating curve; Ref, reference.

## Data Availability

The data presented in this study are available on request from the corresponding author. The data are not publicly available due to institutional policy.
